# Internet-based perioperative exercise program in patients with Barrett’s carcinoma scheduled for esophagectomy [iPEP - study] a prospective randomized-controlled trial

**DOI:** 10.1186/s12885-017-3400-8

**Published:** 2017-06-14

**Authors:** Daniel Pfirrmann, Suzan Tug, Oana Brosteanu, Matthias Mehdorn, Martin Busse, Peter P. Grimminger, Florian Lordick, Torben Glatz, Jens Hoeppner, Hauke Lang, Perikles Simon, Ines Gockel

**Affiliations:** 1 Department of Sports Medicine, Disease Prevention and Rehabilitation, Johannes Gutenberg-University of Mainz, Albert-Schweitzer-Str. 22, D-55128 Mainz, Germany; 2 Clinical Trial Centre Leipzig, University Leipzig, Leipzig, Germany; 3 Department of Visceral, Transplantation, Vascular and Thoracic Surgery, University Hospital of Leipzig, Leipzig, Germany; 4 Institute for Sports Medicine and Prevention, University of Leipzig, Leipzig, Germany; 5 Department of General, Visceral and Transplant Surgery, University Hospital of Mainz, Mainz, Germany; 6 University Cancer Centre Leipzig, University Medicine Leipzig, Leipzig, Germany; 7 Department of General and Visceral Surgery Medical Centre, University of Freiburg, Freiburg, Germany

**Keywords:** Perioperative, Internet-based, Oesophageal cancer, Exercise

## Abstract

**Background:**

Patients undergoing surgery for esophageal cancer have a high risk for postoperative deterioration of lung function and pulmonary complications. This is partly due to one-lung ventilation during thoracotomy. This often accounts for prolonged stay on intensive care units, delayed postoperative reconvalescence and reduced quality of life. Socioeconomic disadvantages can result from these problems. Physical preconditioning has become a crucial leverage to optimize fitness and lung function in patients scheduled for esophagectomy, in particular during the time period of neoadjuvant therapy.

**Methods/Study design:**

We designed a prospective multicenter randomized-controlled trial. The objective is to evaluate the impact of an internet-based exercise program on postoperative respiratory parameters and pneumonia rates in patients with Barrett’s carcinoma scheduled for esophagectomy. Patients are randomly assigned to either execute internet-based perioperative exercise program (iPEP), including daily endurance, resistance and ventilation training or treatment as usual (TAU). During neoadjuvant therapy and recovery, patients in the intervention group receive an individually designed intensive exercise program based on functional measurements at baseline. Personal feedback of the supervisor with customized training programs is provided in weekly intervals.

**Discussion:**

This study will evaluate if an intensive individually adapted training program via online supervision during neoadjuvant therapy will improve cardiorespiratory fitness and reduce pulmonary complications following esophagectomy for Barrett’s cancer.

**Trial registration:**

NCT02478996, registered 26 May 2015.

## Background

Risk management in esophageal surgery has become an essential feature with regard to postoperative outcome [1]. Although perioperative mortality in esophageal cancer surgery could be steadily reduced in recent years, morbidity is still high, and affects 30–50% of patients – even in the current literature and large surgical centers [2]. Pulmonary complications following esophagectomy are the most common and cost-intensive adverse events, mostly due to prolonged intensive care unit stays. This is partly due to one-lung ventilation (OLV) during thoracotomy. During OLV, the operated lung remains completely atelectatic. The non-ventilated lung is known to be hypoperfused owing to hypoxic vasoconstriction, and when the bronchial block is ended, the ensuing immediate lung re-expansion along with tissue reperfusion generates oxygen-derived free radicals. The single-ventilated lung is subjected to a combination of hyperoxia, volutrauma, and hyperinflation. Esophageal complications are not only life-threatening and associated with a high mortality in the short-term postoperative course, but have also exerted a long-lasting negative effect on Health-Related Quality of Life (HRQL) in patients who survive 5 years after esophagectomy for cancer [3]. Advances in surgical techniques, such as minimally-invasive esophagectomy (MIE), have been proposed to reduce surgical risk and perioperative morbidity and mortality, thus improving surgical short- and long-term outcomes. However, to the best of our knowledge, there is only one fully published prospective randomized trial comparing minimally-invasive versus conventional / open surgery (“time trial” = “**t**raditional **i**nvasive vs. **m**inimally-invasive **e**sophagectomy”) [4]. In this study, a significantly reduced rate of pulmonary infections in patients receiving minimally-invasive treatment versus open surgery was reported [4]. This striking result after 2 weeks following surgery might be due to the reduced trauma of thoracoscopy compared to thoracotomy. In addition, oncologic parameters, such as clear resection margins, and the number of dissected lymph nodes, were comparable in both groups [4]. As impressively shown for colorectal surgery, “fast-track” (FT) rehabilitation or enhanced recovery after surgery (ERAS) programs in visceral surgery, comprising atraumatic surgical techniques, stress-reduction of the organism as a response to major surgery, and a decrease of postoperative organ dysfunction, leads to fewer perioperative complications, while reducing hospital costs and increasing patient turnover by shorter lengths of in-hospital stay [5, 6]. However, there are only few studies with limited case numbers regarding fast-track concepts in esophageal surgery [7–12]. Although no routine use of minimally-invasive surgery and other advances of modern surgical techniques were applied, all studies revealed a very short ICU stay, early extubation, thoracic peridural anaesthesia, multimodal pain management, early enteral nutrition (usually via a jejunal catheter), enhanced postoperative mobilisation, and physio−/ ventilation therapy [7–12]. Fast-tracking or enhanced recovery programs for esophagectomy are promising, but still far from reaching the results of e.g. colorectal or minor non-oncological surgery, owing to the higher invasiveness of the procedure with the associated increased general and surgical risk.

A recently published systematic review concluded that preoperative exercise therapy can be effective for reducing postoperative complication rates and lengths of hospital stay after cardiac and abdominal surgery [13]. Preoperative physical activities were lower in patients who developed postoperative pulmonary complications in a study by Feeney et al. [14]. Taking into account the importance of the physical condition, the preoperative physical activity level should be further examined [14]. A prospective observational study by Tatematsu et al. confirmed low-level physical activity – among others – as a significant risk factor for postoperative complications after esophagectomy [15].

One randomized controlled pilot study [16], two non-randomized controlled pilot studies [17, 18], one retrospective cohort study [19], and one study protocol [20] are available in the current literature with regard to preoperative inspiratory muscle training and postoperative pulmonary complications in patients undergoing esophagectomy. In all studies, preoperative inspiratory muscle training was feasible and effective as it resulted in a reduced postoperative pulmonary complication rate [16–19]. Following these encouraging results, we designed an internet-based exercise program for patients scheduled for esophagectomy. The objective of this study is to investigate the pulmonary outcomes of physically preconditioned patients with Barrett’s cancer scheduled for esophagectomy who receive physical training during the phase of neoadjuvant therapy.

## Methods/Study design

This is a prospective multicenter randomized-controlled trial organized by the Department of Sports Medicine, Disease Prevention and Rehabilitation of the Johannes Gutenberg-University of Mainz, Germany, and the Department of Visceral, Transplant, Thoracic and Vascular Surgery of the University Medical Center of Leipzig, Germany. Patients are randomly assigned to either undergo internet-based perioperative exercise program (iPEP) (intervention group) or treatment as usual (TAU) (control group). The randomization procedure is performed using an online random number generator, which is provided by the University of Geneva’s online service [21]. Randomization is stratified by center. The study is funded by the “Barrett-Initiative e.V.” (http://www.barrett-initiative.de/). Inclusion (Table 1) and exclusion (Table 2) criteria are clearly defined. Primary staging, neoadjuvant treatment, preoperative nutrition as customized to the initial nutritional risk screening (NRS) [22], re-staging, preoperative peridural anesthesia, abdomino-thoracic esophagectomy with 2-field lymph node dissection and intrathoracic anastomosis, postoperative fluid management and nutrition, analgesia, physical therapy, mobilization and ventilation therapy following esophagectomy as well as rehabilitation are performed according to standard operating procedures (SOPs). At the beginning of the study, smokers are advised and supported to stop smoking by smoking cessation programs when needed. Smoking habits are recorded throughout the study.

**Table 1:** Inclusion criteria

#	Inclusion criteria
1.	Histologically proven adenocarcinoma of the esophagus or adenocarcinoma of the esophagogastric junction type I-II according to Siewert’s classification, clinical stages IIB-IIIC (T3/T4 and/or N+; M0) according to UICC, 7th edition
2.	Male
3.	Age between 18 and 75 years
4.	Resectable stage according to discussion in the local multidisciplinary tumor board (MDT) of the participating centers and patient medically fit for multimodality therapy (ECOG performance status at least 1 or better, no severe impairment of cardiac, renal, hepatic, endocrine, bone marrow and cerebral functions)
5.	Planned abdomino-thoracic esophagectomy with gastric pull-up and intrathoracic or cervical anastomosis
6.	Cognitive ability of the patient to understand the perioperative program and to participate actively [40, 41]

**Table 2:** Exclusion criteria

#	Exclusion criteria
1.	Presence of a second malignant tumor (unless curatively treated >5 years ago)
2.	Chemotherapy or radiochemotherapy in patient’s history
3.	Orthopedic, rheumatologic, cardiovascular or neurologic (epilepsy, stroke, Parkinson’s disease, muscle wasting diseases such as amyotrophic lateral sclerosis or multiple sclerosis) contraindications for the exercise program
4.	Inability to use the internet or no internet access
5.	Inability to communicate in German
6.	Each active disease, which hinders completion of the study
7.	Active alcoholism or illegal drug consumption within the last six months before study entry

Primary objective of the trial is to investigate the effect of the perioperative training on physical capacity.

Primary endpoint is the change in peak oxygen uptake (VO_2peak_) prior to surgery as compared to baseline.

Secondary endpoints are

- Change in VO_2peak_ at 12 weeks after surgery
- Change in Forced Expiratory Volume in 1 s (FEV1) directly prior to surgery and at 12 weeks after surgery
- Change in Forced Vital Capacity (FVC) directly prior to surgery and at 12 weeks after surgery

All respiratory parameters are assessed by a spirometer.

Further secondary endpoints encompass pneumonia within 12 weeks after surgery, anastomotic insufficiency within 12 weeks after surgery (according to Veeramootoo et al.) [23], duration of mechanical ventilation, re-intubation rate, length of intensive care unit stay, postoperative in-hospital stay, feasibility of the internet-based exercise program, quality of life as assessed with the EORTC (European Organization for Research and Treatment of Cancer), QoLQ-C30 questionnaire with the esophagus-specific module OES-18 [24], and social support of disease coping by means of the modified *Berlin Social Support Scal*e [25].

### Sample-size calculation and statistical analyzes

Our assumptions for sample size calculation are based on reports investigating the effect of different training modalities on respiratory parameters in cancer patients during or after therapy [26–31]. We aim at detecting a difference in mean VO_2peak_ of 6 ml/min/kg between the two groups. This difference seems to be achievable, given the reports mentioned above. The reported standard deviation (SD) VO_2peak_ ranges between 2.3 and 10.7 ml/min/kg. For our trial, we rather conservatively assume a SD of 8 ml/min/kg. With these assumptions, a total of 58 patients (29 interventions and 29 controls) have to be analyzed to achieve a power of 80% at a significance level of 5% (two-group t-test). Because the primary endpoint is assessed by spiroergometry and this may be burdensome for patients, we estimate a rather high dropout-rate of about 30%. Taking this into account, a total of 80 participants (40 interventions and 40 controls) will be randomized.

Primary efficacy analysis will be performed by ANCOVA with baseline values of VO_2peak_ as covariates, thus increasing power. Confirmatory analysis will be based on all patients with available post-baseline VO_2peak_ measurement. Patients will be analyzed as randomized.

### Intervention

Before initial spiroergometry and echocardiography, participants will perform a breathing test, which provides information about actual inspiratory vital capacity. The patients perform a stepwise cardiopulmonary exercise test on a treadmill until exhaustion (Table 3). The test includes up to 14 stages and is increased by speed and angle of inclination. Termination of ergometry is indicated by meeting absolute or relative stop criteria (Table 4) [32].

**Table 3:** Modified “walking protocol”

Stage	Speed (kilometers/hour)	Angle of inclination (degree)	Duration (minutes)
1	3	1.5	3
2	3.7	3.0	3
3	4.4	4.9	3
4	5.1	6.3	3
5	5.8	7.4	3
6	6.5	8.2	3
7	6.5	9.8	3
8	6.5	11.4	3
9	6.5	13.0	3
10	6.5	14.6	3
11	6.5	16.2	3
12	6.5	17.8	3
13	6.5	19.4	3
14	6.5	21.0	3

**Table 4:** Absolute and relative criteria indicating termination of ergometry [31]

Absolute indication	Relative indication
ECG ST-segment depression ≥3 mm	Hypertensive dysregulation (RR_syst_ 230–260 mmHg, RR_diast_ ≥ 115 mmHg)
ECG ST-segment elevation ≥1 mm	Drop in blood pressure > 10 mmHg (compared to baseline blood pressure) without signs of myocardial ischemia (no angina pectoris, no ECG ST-segment depression)
Drop in blood pressure > 10 mmHg (compared to baseline blood pressure) with signs of myocardial ischemia (angina pectoris, ECG ST-segment depression)	Polymorphic extrasystols, duplets (2 consecutive ventricular extrasystols), salves (≥ 3 consecutive ventricular extrasystols)
Moderate-severe angina pectoris-symptoms	Supraventricular tachycardias
Severe dyspnea	Bradyarrhythmias
Clinical signs of less perfusion (cyanosis)	Line faults
Persistent (duration >30 s) ventricular tachycardias	Presence of line faults (high AV-blockage, branch block)
Exhaustion of the patient	reinforced angina pectoris symptoms
Technical problems (defect ECG-registration, monitor failure)	

### Internet-based perioperative exercise program (iPEP)

After randomization, the iPEP group is supervised by a Sports Scientist before and after surgery. Information and training on how to handle the webpage as well as supervision throughout the study is provided by one and the same person (D.P.). All crucial steps interacting with the homepage are written down in the Study Manual (SM). Patients are delivered a heart rate monitor (“*pulse watch*”) and a Thera-Band® for further mobilization and training free of charge. In parallel with the neoadjuvant therapy, patients receive an individually designed intensive exercise program based on the functional and fitness measurements at first diagnosis (3 months). Personal feedback of the supervisor with adapted and potentially accelerated training programs is provided in weekly intervals via Email function. After each training session patients have to complete different medical and exercise-specific information as unmodified BORG scale value, resting heart rate, maximum heart rate, recovery pulse and symptoms / problems experienced during exercise in a table important for training control [33]. During hospitalization for esophagectomy and further rehabilitation, participants can continue to use the functions “*Chat*” and “*Forum*” of the webpage. As soon as returned home, postoperative supervision by exercise program continues for another 3 months, similar to the preoperative phase. Thus, levels of training are reduced and customized to the potentially lower proficiency following major surgery. Progressive increase of training is intended to restore full reconvalescence. In case of problems, a “*Live Chat*” is offered in regular intervals for direct communication between trainer and patient. Acute problems can be addressed to the supervisor at any time and will be answered within 24 h.

### Training

**Fig. 1:**
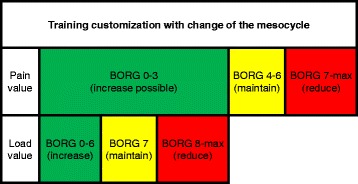
Criteria of training customization

Because of the short intervention time and in order to achieve an optimum development of the physical resources until surgery, daily exercise is advised consisting of a mixture of endurance, resistance and ventilation training. The concept follows the principle of periodizing and cyclizing. Interventions during 3 month phases each - before and after esophagectomy - are divided into macro- (= 3 months), meso- (= 1 month) and microcycles (= 1 week). The latter can only be differentiated by one training unit. Increase in training frequency is intended each mesocycle, according to potential progress in performance. Individual adaptation of the exercise program can be performed after each microcycle. The relationship between the symptoms / problems the patient experiences during training and the subjective assessment of efforts with regard to criteria of program adaptations is demonstrated in (Fig. 1 **)**.

Endurance training is increased by frequency of units, extension of duration and enhanced intensity of each unit (extensive and intensive interval load). Resistance training is provided by easily feasible exercises with minimal potential for error. Eleven strength exercises should be carried out in a prescribed sequence, in order to stimulate muscular strength in the major muscle groups of the upper part of the body. Neck press, butterfly, different variations of rowing, and lat exercises will be carried out with the elastic band. At the end of the training session, patients are encouraged to perform stretching and breathing exercises for relaxation and a conscious breathing. An illustrated instruction and video files for the exercises are available on the website. Alterations shall not be made without personal feedback and re-assessment. An individualization of the training is achieved, adapting the number of repetitions, number of sets or the use of a stronger band.

In addition, daily ventilation exercises are carried out with the “*Voldyne*” device. Every day, one set with ten repetitions must be performed and the maximum value should be noted in the training schedule.

### Treatment as usual (TAU)

Participants of the TAU group receive written information material enlightening the importance of regular physical activities and general releases on preparation for esophagectomy. All interviews (such as medical history; nutritional risk score; EORTC-QLQ-C30 & EORTC-QLQ-OES18; international physical activity questionnaire (IPAQ)), examinations (echocardiogram; spirometry), and fitness tests (spiroergometry), during the study period as well as surgery and perioperative management were standardized in accordance to the intervention group. The TAU group is also advised to quit smoking, if necessary. However, the TAU group does not receive any further advice based on their individual fitness testing. Instead, they are advised to be active for up to 150 min a week in accordance to the exercise guidelines for cancer survivors [34].

### Time course of measurements

Investigations are performed at 3 different time points of the study: 1.) t0 = baseline at first diagnosis, 2.) t1 = after neoadjuvant treatment, immediately before surgery, and 3.) t2 = follow-up 3 months after esophagectomy.

**Fig. 2:**
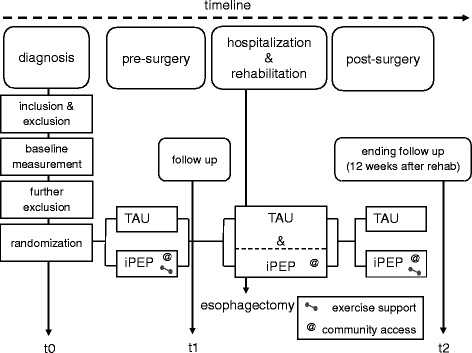
Time course of interventions (iPEP and TAU)

The flow chart in Fig. 2 depicts the time course of iPEP and TAU with respective points of measurements.

### Translational research

Blood (t0-t2) and tumor tissue samples (t0, t1 and surgical specimen) will be stored to enable translational research and assess potential predictive factors of outcomes.

## Discussion

In spite of significant advances in surgical techniques, postoperative intensive care and complication management, esophagectomy is still a high risk-procedure associated with considerable postoperative pulmonary morbidity and mortality. This often encounters for prolonged periods of reconvalescence and reduced quality of life, apart from socioeconomic disadvantages. In addition, postoperative pulmonary complications after esophagectomy are associated not only with worse short-, but also long-term outcomes [35]. Although morbidity and functional results following esophagectomy are known to be multifactorial, regular, structured physical activity and ventilation training before surgery seem to be the most relevant leverages to optimize results [13–20]. As most patients with locally advanced, potentially resectable esophageal cancer undergo neoadjuvant treatment, this period offers the possibility for improving fitness, even in patients with no or only few previous physical activities. As shown by a recent metaanalysis, neither neoadjuvant chemotherapy nor radiochemotherapy for esophageal carcinoma increases the risk of postoperative morbidity or perioperative mortality compared with surgery alone [36]. There was also no clear difference between the two neoadjuvant treatment modalities with respect to postoperative morbidity [36]. Therefore, both modalities are allowed in our study. The “*Nutrition and Physical Activity Guidelines for Cancer Survivors*” recommend a minimum of 150-min exercise per week, and strength training at least 2 days per week [37]. Because of the short intervention time and in order to achieve an optimum development of physical resources until surgery, a daily training is advised in our study. Participants should perform up to four units of endurance and up to 3 units of resistance training per week (approximately one hour per unit) in the last phase of training [38]. In addition, daily ventilation exercises are carried out with the “*Voldyne*” device [39].

To our knowledge, this is the first prospective randomized controlled trial to assess an internet-based perioperative setting for patients scheduled for esophagectomy.

To ensure individual extensive care, the use of our online exercise program is easily accessible.

This novel concept might have several advantages: (i) the patients can perform exercises at home, (ii) individual, close and stepwise adaptation of the program is possible according to patient’s actual status, physical efficacy and symptoms / side effects of tumor presence or neoadjuvant therapy, (iii) there is only one supervisor / trainer for all centers of the study, strongly reducing the potential of bias due to different caregivers involved, (iv) social networking of all participants via different channels of communication will be possible in order to increase compliance and motivation to complete the program, and (v) an “open” area of the webpage offers information and news regarding Barrett’s carcinoma and respective measures / therapies, which can also be used by partners, relatives and friends of the individual. All these aspects supply the possibility to enable consequent and close care with the overall aim to reach a maximum of physical fitness and lung function before surgery. This also bears the potential for durable and healthy lifestyle changes and is enforced by the fact that patients can use the webpage even beyond their cancer treatment and study participation. Through active engagement with other patients the social factor is positively affected.

To avoid unwanted guests, we have constructed a private login area and the supervisor sets as administrator the accounts of the patients at study entry. Hence, a login without participation in the study is impossible.

The objective of our study is to evaluate for the first time the impact of an internet-based perioperative exercise program in patients with Barrett’s carcinoma scheduled for esophagectomy on respiratory parameters and pneumonia rates. Based on our concept, we hypothesize that the postoperative course of patients might be less complicated while functionally optimized. This multicenter study has started recruitment. Centers meeting the logistics of this study are welcome to participate and contact the address for correspondence (PS) for the detailed study protocol, Study Manual (SM) and Case Report Forms (CRFs).

## Abbreviations

CRF: Case report form
EORTC: European organization for research and treatment of cancer
ERAS: Enhanced recovery after surgery
FEV1: Forced expiratory volume in 1 s
FT: Fast-track
FVC: Forced vital capacity
HRQL: Health-related quality of life
ICU: Intensive care unit
iPEP: internet-based perioperative exercise program
MIE: Minimally-invasive esophagectomy
NRS: Nutritional risk screening
OLV: One-lung ventilation
SM: Study manual
SOP: Standard operating procedures
TAU: Treatment as usual
VO_2peak_: Peak oxygen uptake
